# Multi-omics analysis of macrophage-associated receptor and ligand reveals a strong prognostic signature and subtypes in hepatocellular carcinoma

**DOI:** 10.1038/s41598-024-62668-x

**Published:** 2024-05-28

**Authors:** Yulou Zhao, Cong Chen, Kang Chen, Yanjun Sun, Ning He, Xiubing Zhang, Jian Xu, Aiguo Shen, Suming Zhao

**Affiliations:** 1 Department of Interventional and Vascular Surgery, Affiliated Hospital of Nantong University, Nantong, China; 2 Medical School, Nantong University, Nantong, China; 3 The Sixth People’s Hospital of Yancheng City, Yancheng, China; 4 Department of Medical Oncology, Nantong Second People’s Affiliated Hospital of Nantong University, Nantong, China; 5 Cancer Research Center Nantong, Affiliated Tumor Hospital of Nantong University, Nantong, China

**Keywords:** Hepatocellular carcinoma 1, Macrophage 2, Receptor and ligand 3, Immunotherapy 4, Single-cell analysis 5, Prognostic signature 6, Hepatocellular carcinoma, Tumour biomarkers, Cancer genomics

## Abstract

Hepatocellular carcinoma (HCC) is a significant contributor to morbidity and mortality worldwide. The interaction between receptors and ligands is the primary mode of intercellular signaling and plays a vital role in the progression of HCC. This study aimed to identify the macrophage-related receptor ligand marker genes associated with HCC and further explored the molecular immune mechanisms attributed to altered biomarkers. Single-cell RNA sequencing data containing primary and recurrent samples were downloaded from the China National GeneBank. Cell types were first identified to explore differences between immune cells from different sample sources. CellChat analysis was used to infer and analyze intercellular communication networks quantitatively. Three molecular subtypes were constructed based on the screened twenty macrophage-associated receptor ligand genes. Bulk RNA-Seq data were downloaded from The Cancer Genome Atlas and Gene Expression Omnibus databases. After the screening, the minor absolute shrinkage and selection operator (LASSO) regression model was employed to identify key markers. After collecting peripheral blood and clinical information from patients, an enzyme-linked immunosorbent assay (ELISA) was used to detect the correlation between key markers and IL-10, one of the macrophage markers. After developing a new HCC risk adjustment model and conducting analysis, it was found that there were significant differences in immune status and gene mutations between the high-risk and low-risk groups of patients based on macrophage-associated receptor and ligand genes. This study identified SPP1, ANGPT2, and NCL as key biological targets for HCC. The drug-gene interaction network analysis identified wortmannin, ribavirin, and tarnafloxin as potential therapeutic drugs for the three key markers. In a clinical cohort study, patients with immune checkpoint inhibitor (ICI) resistance had significantly higher expression levels of OPN, ANGPT2, NCL, and IL-10 than patients with ICI-responsiveness. These three key markers were positively correlated with the expression level of IL-10. The signature based on macrophage-associated receptor and ligand genes can accurately predict the prognosis of patients with HCC and the sensitivity to immunotherapy. These results may help guide the development of targeted prevention and personalized treatment of HCC.

## Introduction

Globally, hepatocellular carcinoma (HCC) accounts for 75–85% of instances of primary liver cancer. HCC was the third most common cancer-related cause of death globally in 2020 and the sixth most prevalent cancer overall^1^. For HCC, surgery is the preferred course of action. However, the cancer has often already progressed to the middle to late stages when detected, and the window for surgery is usually missed. Palliative therapies, such as transarterial chemoembolization (TACE), radiation therapy, local ablation, targeted drugs, immunotherapy, and a combination of these methods are mainly used for unresectable HCC. Still, these are only minimally effective^2,3^. As a result, the use of immunotherapeutic drugs to treat HCC has gradually increased in recent years. In 2020, the Chinese National Medical Products Administration (NMPA) and the U.S. Food and Drug Administration (FDA) approved the first-line HCC treatment, atezolizumab plus bevacizumab. However, immunotherapy only helps a small percentage of patients, and some experience major side effects or relapse within a short amount of time^4^. Exploring new and valuable markers for immunotherapy and the predictive monitoring of HCC is thus urgently needed.

Numerous solid tumors can be successfully treated with immunotherapy that targets the immune checkpoint system, and some studies have indicated that treating HCC with immunotherapy has promising therapeutic outcomes^5–7^. Immunological checkpoints can deactivate immunological responses in the tumor microenvironment (TME), such as PD-1/PD-L1 (programmed cell death protein 1/programmed cell death ligand 1)^8^, and immune checkpoint inhibitors (ICIs), such as anti-PD1/PD-L1 antibodies are clinically effective in treating various solid malignancies. Tumor-associated macrophages (TAMs) enter tumor tissues and can suppress immune system activity, promote tumor cell growth, and regulate tumor cell metabolism^9–11^. TAMs induce PD-L1 expression via IL-1β signaling in HCC cells^12^. However, colony-stimulating factor-1 (CSF-1) signaling induced by bone bridge protein (OPN) can block TAM transport, which makes HCC sensitive to PD-L1 blockade^13^. It has been shown that one significant TAM-mediated mechanism implicated in immunosuppression is the expression of T-cell immune checkpoint ligands (such as PD-L1) in TAM^14^, and TAM signaling through receptor ligands may therefore play a decisive role in the effects of immunotherapy^15^. However, only a few studies have looked at the molecular classification of HCC patients based on genes that bind to the macrophage receptor, and we think that this is crucial for screening potential populations and creating individualized and preventive immunotherapy plans.

In this work, we performed a cellular communication analysis to discover macrophage-associated receptor ligand gene sets using single-cell sequencing data from primary HCC and relapsed HCC. One-way Cox regression analysis was used to find prognosis-related regulators, whereas univariate Cox regression analysis was utilized to find prognosis-related genes and build molecular subtypes. The molecular subtypes were then constructed based on these genes. A validated prognostic risk signature for HCC was subsequently constructed based on differentially expressed genes (DEGs) among subgroups. We collected and analyzed peripheral blood from patients with HCC who had received immunotherapy to look for connections with the efficacy of the treatment and validate the therapeutic use of the core genes in the signature. We anticipate that the results of the current study will provide targeted prevention and personalized medical care that improves survival outcomes and reverses the immune resistance profile in HCC.

## Materials and methods

### Dataset collection and preprocessing

The mRNA gene expression profile data (TPM format) of HCC patients were retrieved from The Cancer Genome Atlas (TCGA) (https://portal.gdc.cancer.gov) and Gene Expression Omnibus (GEO) (https://www.ncbi.nlm.nih.gov/geo/) databases. The grounds for data exclusion were the lack of complete follow-up information, survival of 0 days, and repeat sequencing in the same patient. Data on somatic cell mutations were acquired from the TCGA database for 371 tumor samples having copy number variations (CNV). Mutation waterfall plots were visualized using the Maftools R package^16^. The CNP0000650 dataset was downloaded from the China National GeneBank (CNGB) (https://db.cngb.org/cnsa) to conduct single-cell analyses, and six recurrent and 12 primary samples from the dataset were included in this study. To homogenize the gene expression data from all of the RNA-seq data, ComBat^17^ was employed. We downloaded image files with immunohistochemistry analysis from Protein Atlas (http://www.proteinatlas.org).

### Single-cell sequencing data analysis

Genes that were expressed in < 3 Cells were excluded, as were cells containing < 300 expressed genes. The percentage mitochondrial transcript expression was calculated using each cell's percentage feature set function. To ensure high-quality analysis, we retained cells containing more than 100 unique molecular identifiers (UMIs), 100–5000 expressed genes, and a mitochondrial content lower than 5%. In our next step, we normalized the merged data and identified highly variable genes using the FindVariableFeatures function [which identifies variables based on the variance stabilization transformation (‘vst’)]. A principal component analysis (PCA) was then performed using the RunPCA function, and clustering based on the shared nearest-neighbor graphs was conducted using the Seurat functions FindNeighbors and FindClusters (resolution = 0.5). Eighteen subgroups were identified in this study. We used classical marker genes and the singleR package to annotate cells^18^ and obtained macrophages, T cells, NK cells, hepatocytes, B cells, monocytes, neuroepithelial cells, fibroblasts, and  dendritic cells (DCs). CellChat (version 1.4.0) was used to analyze the intercellular communication between different cells.

### Identification of subtypes based on macrophage-associated receptor ligand genes

Among the macrophage-associated receptor genes, predictive genes were found using a single-variable Cox regression analysis. A PCA and an unsupervised consistent cluster analysis were run on the prognostic genes to ascertain whether each subtype was largely independent. The number of clusters was determined using the R package "ConensusClusterPlus," and 100 replicates with pltem = 0.8 were performed to verify the stability of the isoforms. Survival analyses were performed using Kaplan–Meier curves, and the outcomes were evaluated using a log-rank (Mantel–Cox).

### Identification of DEGs

We examined differentially expressed genes between subtypes using the R Bioconductor package limma (V3.46.0)^19^. Genes with an adjusted P-value of 0.05 and |Log2 fold-change (log2FC) |> 1 were classified as differentially expressed. P-values were corrected using the Benjamini-false Hochberg's discovery rate (FDR). With the help of the R software program ggplot2 V3.3.5, volcano plots were produced. R software's Pheatmap V1.0.12 was used to plot the DEG heatmaps from each comparison. A Venn diagram was created in R using the VennDiagram package (https://CRAN.R-project.org/package=VennDiagram).

### Functional enrichment analysis

Analyses of gene ontology (GO) and Kyoto Encyclopedia of Genes and Genomes (KEGG) data were conducted using the R package "clusterProfiler" (https://bioconductor.org/packages/clusterProfiler/). The use of KEGG data has been authorized by the institution, for which we express our gratitude^20–22^. Enrichments were considered significant if associated with a *p* value of < 0.05 and an FDR q-value of < 0.05. GSVA was used for functional enrichment analysis to determine the connections between molecular subtypes and biological functions. The reference gene set for GSVA was C2.cp.kegg.v7.0.symbols.gmt, with an FDR < 0.05.

### Construction and validation of prognostic signature

In this study, the TCGA- Liver Hepatocellular Carcinoma (TCGA-LIHC) cohort was used for modeling, and the Gene Expression Omnibus (GSE14520)^23^ cohort was used for validation. A risk model was developed using the Least Absolute Shrinkage and Selection Operator (LASSO) regression and tenfold cross-validation; this was conducted for 1,000 cycles with 1,000 random iterations to prevent overfitting effects. In the training and external validation cohorts, the predictive value of risk scores was evaluated using univariate and multivariate Cox regression analyses. Time-dependent subject operating characteristic (ROC) curves were used to compare the predictive performance of the risk scores with conventional clinicopathological parameters.

### Immune infiltration and correlation analysis

Immune cell infiltration in the TME was assessed using seven methods: XCELL, TIMER, QUANTISEQ, MCPcounter, EPIC, CIBERSORT, and CIBERSORT-ABS. In all samples, stromal, immune, and ESTIMATE scores were calculated using the ESTIMATE algorithm (version 1.0.13) to reflect the state of the microenvironment^24^. A previous cohort with open information (IMvigor210) was used to gather gene expression data and immunotherapeutic efficacy to assess the prediction performance of risk ratings for the prognosis of patients receiving immunotherapy^25^. Different risk groups were characterized using the immunogenomic features identified by Thorsson et al., and these included wound healing (C1), IFN-γ dominant (C2), inflammatory (C3), lymphocyte depleted (C4), immunologically quiet (C5), and TGF-β dominant (C6) features^26^.

### Drug sensitivity analysis

Drug sensitivity data were downloaded from the Genomics of Drug Sensitivity in Cancer (GDSC) database (www.cancerrxgene.org)^27^ and calculated using the "PRrophytic" R package in R software. The Wilcoxon signed-rank test was applied to see whether the risk groups' IC50 values varied. Box plots are used to display the results.

### Study population and clinical data collection

We enrolled 50 patients who received ICI at the Affiliated Hospital of Nantong University, Nantong, Jiangsu, China, between January 2020 and January 2022. Enzyme-linked immunosorbent Assay (ELISA) was performed on blood samples donated by the participants with their informed consent. The patient inclusion criteria are as follows: (1) pathologically confirmed HCC, (2) immunotherapy with PD-1 inhibitors, (3) patient survival longer than three months, and (4) patients without organic lesions other than HCC. Patient exclusion criteria include (1) recurrent HCC, (2) other treatment during immunotherapy, (3) inability to follow up regularly and missed visits, (4) significant abnormalities in blood or liver and kidney functions that are more than three times the average value, and (5) being pregnant or lactating. Based on the criteria proposed by the Society for Immunotherapy of Cancer (SITC), objective responses determined by the investigator were assessed radiologically using computed tomography (CT) 6 months after the start of immunotherapy^28^. According to the immune-related Response Evaluation Criteria in Solid Tumor (iRECIST)^29^, the ICI resistance group (immune unconfirmed progressive disease (iUPD) and immunological confirmed progressive disease (iCPD)) and the ICI response group (immune complete response (iCR), immune partial response (iPR), and immune stable disease (iSD)) were created from the patient population.

### Human blood sample preparation

Peripheral blood was drawn from the patients six months after the first immunotherapy session, and an ELISA analysis was conducted. Under aseptic conditions, peripheral blood was mixed with Hank’s buffer (Sigma, H8264) at a 1:1 ratio (4 mL:4 mL). Centrifuge tubes (15 mL) were marked, and 3 mL of human lymphocyte isolate (Sigma, 10771–100 ML) was added to each tube. Subsequently, 6 mL of diluted blood was gently added to the upper layer of the lymphocyte isolate. The centrifuge tubes were then placed in a centrifuge for 20–25 min (400 g, 25 °C), and the middle cloudy layer was carefully aspirated with the peripheral blood mononuclear cells (PBMC) and placed in a 15 mL tube. PBS at a volume of approximately 2–3 mL, the same as that of the PBMC-containing liquid, was then added to the centrifuge tube, which was centrifuged for 5 min (180 g, 25 °C). The supernatant was discarded, 1 mL of lyophilization solution was added, and the tube was stored at − 80 °C until use. Subsequently, 3–4 mL of blood was centrifuged for 15 min (3000 rpm, 25 °C), and the supernatant was frozen at − 80 °C until use.

### Enzyme-linked immunosorbent assay (ELISA)

The PBMC was thawed from − 80 °C and centrifuged for 5 min (13,000 rpm, 4 °C) to enable the detection of NCL and IL-10. After removing the supernatant, the sediment was suspended in 20 μL RIPA cracked buffer (Beyotime, P0013B) and kept on ice for 10 min. Frozen serum was removed and thawed at room temperature to enable the detection of OPN and ANGPT2. The levels of NCL (Mlbio, ml038241), IL-10 (Mlbio, ml064299), OPN (Mlbio, ml038278), and ANGPT2 (Multi Sciences, EK1215) were measured using ELISA kits according to the manufacturer’s protocol. Samples and standards were tested in triplicate. After terminating the enzyme–substrate reaction, the optical density (OD) of the complexes was measured at 450 nm using an enzyme calibrator (MultiskanFC, ThermoScientific), and the concentration of each protein in the blood samples was calculated using a specific standard curve. The Affiliated Hospital of Nantong University's Ethics Review Committee approved this study (ethical review number 2020-K090-01).

### Analysis of drug-gene interactions and molecular docking

First, drug studies related to secreted phosphoprotein 1 (SPP1), angiopoietin 2 (ANGPT2), and nucleolin (NCL) were searched using Pubchem (https://pubchem.ncbi.nlm.nih.gov/), Drugbank (https://www.drugbank.com/), and the Therapeutic Target Database (TTD). The SDF format of compounds was obtained from the PubChem data numbers and subsequently imported into ChemDraw 3D (Version 22). Energy minimization was conducted using the MM2 module to obtain the lowest energy advantage concept, which was saved as a mol2 file. Human SPP1, ANGPT2, and NCL proteins were retrieved from the UniProt (https://www.uniprot.org/) database. The Protein Data Bank identifications (PDBIDs) corresponding to the four genes were 3cxd, 4jzc, and 2krr, which were retrieved from the Protein Data Bank (PDB) database (http://www.rcsb.org/pdb/home/home.do). Visualization was performed separately using PyMOL (version 1.7.2), and MGLTools (Version 1.5.6) was used for dehydration, hydrogen addition, charge calculation, and merging of non-polar hydrogens. The ligand was docked to the receptor using Autodock vina (Version 1.1.1) with the docking parameters shown in Table S1, and the higher-scoring conformation was visualized using Discovery Studio 2021.

### Ethics statement

The study was conducted according to the guidelines of the Declaration of Helsinki and approved by the Ethics Committee of the Affiliated Hospital of Nantong University (Grant No. 2022-K131-01, approval date: 21 June 2022).

## Results

### Single-cell clustering dimension reduction and cell annotation

**Figure 1:**
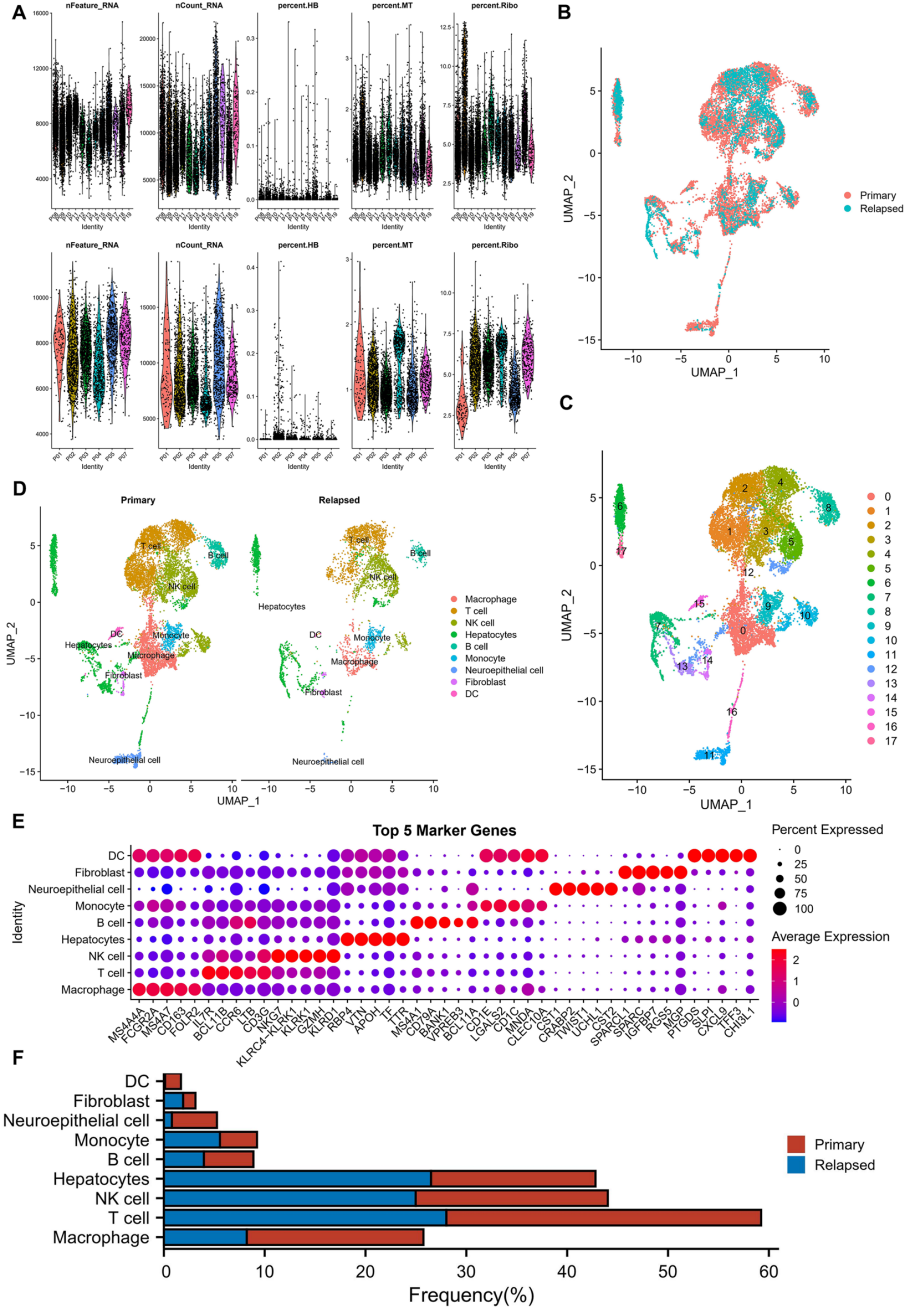
Single-cell clustering dimension reduction and cell annotation. (**A**) Violin plots of gene number (nFeature_RNA), transcript number (nCount_RNA), erythrocyte gene percentage (percent. HB), mitochondrial gene percentage (percent. MT), ribosome gene percentage (percent. Ribo) for each sample after single-cell data filtering. Primary HCC (up), relapsed HCC (down). (**B**). UMAP plots of the distribution of primary and relapsed HCC samples. (**C**) UMAP plots of the distribution of 17 cell subpopulations. (**D**) UMAP plots of each cell subpopulation after annotation. (**E**) Point plots of the top 5 marker genes expression of the subpopulations after CNV annotation. (**F**) Proportions of the cell subpopulations after CNV annotation in each sample.

Single-cell data were filtered using the techniques above by configuring each gene to be expressed in a minimum of three cells and at least 300 genes to be expressed in each cell. The percentages of mitochondria and rRNA were calculated using the PercentageFeatureSet function. We ensured that each cell expressed more than 100 but less than 5000 genes and that each cell's mitochondrial content and UMI were less than 25% and greater than 100, respectively. Ten thousand five hundred forty-eight cells were obtained from primary HCC (12 samples) and 3688 from relapsed HCC (6 samples). Figure 1A shows the violin plot associated with quality control. Data from each of the 18 samples were then normalized by log-normalization. All genes were scaled using the ScaleData function, and PCA downscaling was carried out using RunPCA to find the anchor points. Highly variable genes were found using the FindVariableFeatures function(Fig. 1B). A total of 18 subgroups (Fig. 1C,D) were obtained after clustering the cells using FindNeighbors and FindClusters functions (resolution = 0.5). The cells were annotated using the classical marker genes and singleR package. Nine cell subpopulations of macrophages, T cells, natural killer cells (NK cells), hepatocytes, B cells, monocytes, neuroepithelial cells, fibroblasts, and DC were obtained. The bubble diagram (Fig. 1E) shows the differences between expression levels of the Top 5 genes within the subpopulations, and the bar chart shows the percentages and cell counts of the annotated subpopulations in each sample. The percentage of DC varied significantly, with only 0.11% in the recurrent samples compared to 1.58% in the primary samples (Fig. 1F).

### CellChat analysis conducted to build a cell communication network

**Figure 2:**
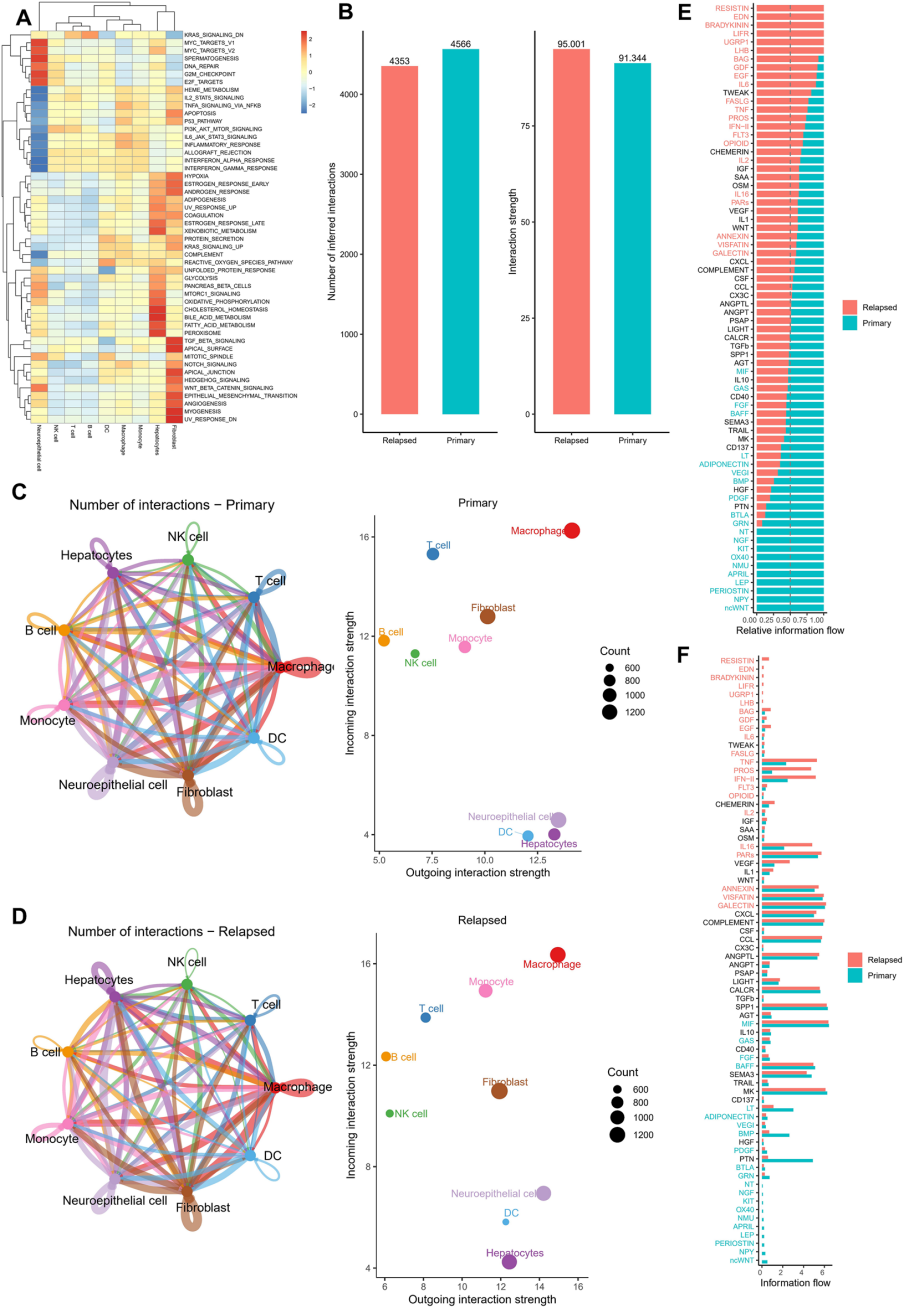
Single-cell enrichment analysis and cell communication network construction. (**A**) Heatmap of pathways significantly enriched by cell type. (**B)** Comparison between primary and relapsed HCC samples in terms of the number and intensity of intercellular communication. (**C**,**D**) Number and strength of common intercellular interactions, primary HCC (**C**), relapsed HCC (**D**). (**E**) Detection of secretory signals in primary and relapsed HCC samples. (**F**) Comparison of secretory signal differences between primary and relapsed HCC samples.

Pathways significantly enriched for each cell type were characterized using heat maps (Fig. 2A). Selected scRNA-seq data were utilized to infer intercellular communication using the CellChat program to investigate the relationships between the identified cells further. Signaling networks have developed to translate internal and external inputs into important cellular choices, such as growth, differentiation, and death^30^. Furthermore, the combined analysis using CellChat provides the opportunity to identify significant signaling changes that may lead to the recurrence and metastasis of HCC. The results showed increased strength of the inferred intercellular interactions in relapsed HCC. However, the number of interactions was decreased compared to those in primary HCC (Fig. 2B). CellChat was also used to infer the outgoing and incoming communication patterns of secretory cells. Based on the strength of the afferent/efferent interactions, the cells in the PRIMARY and RELAPSED groups were mapped onto a shared two-dimensional flow shape (Fig. 2C,D). The results showed that the interaction intensity and number of macrophages in primary and relapsed HCC were significantly stronger than in other cells. However, compared with primary HCC, the outgoing signal of macrophages was further enhanced in relapsed HCC. In addition, multiple unique secretory signals, such as RESISTIN, END, BRADYKININ, LIFR, UGRP1, and LHB, were detected in nine cell populations of the relapsed samples (Fig. 2E). Comparisons between the samples showed that secretory signals such as TNF, PROS, IFN-II, and IL-16 were up-regulated in recurrent samples (Fig. 2F). These findings imply that primary tumors and metastases have dramatically different intercellular communication.

### Construction of molecular subtypes based on macrophage-related receptor ligand genes

**Figure 3:**
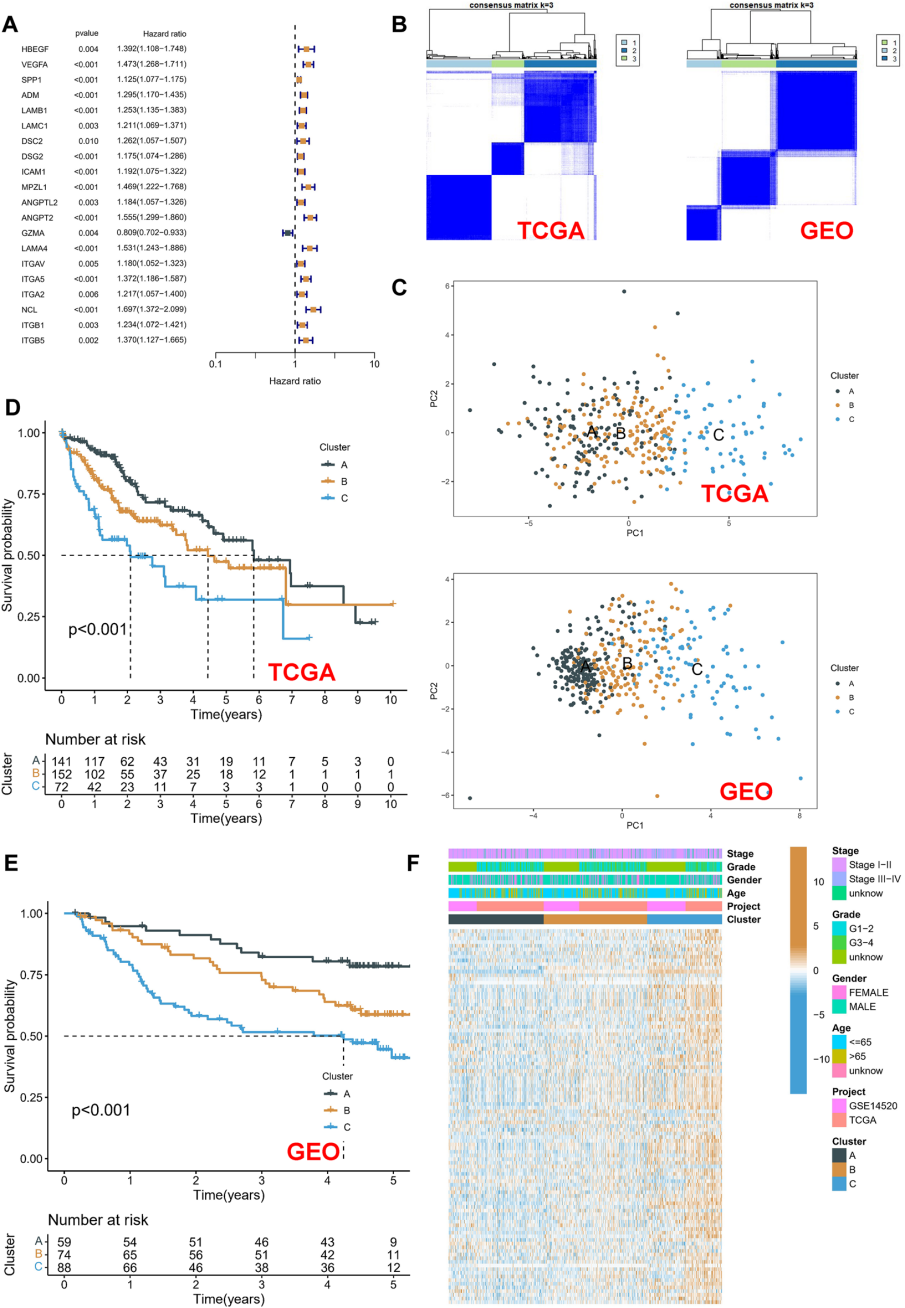
Construction of molecular subtypes based on macrophage receptor ligand genes. (**A**) Univariate Cox regression to identify moderators associated with prognosis. (**B**) Clustering analysis of TCGA-LIHC and GSE14520 patients based on the screened regulatory genes using the NMF algorithm. (**C**) The PCA of clusters demonstrates good heterogeneity. (**D**,**E**) Kaplan–Meier survival curves of OS in three clusters. TCGA-LIHC cohort(**D**), GSE14520 cohort(**E**). (**F**) Heatmap of the correlation between clinical characteristics and gene expression for the three subgroups.

These findings imply that interactions between macrophages and other cells significantly influence the development and spread of HCC. We then conducted further studies on macrophage-associated receptor ligands. 113 macrophage-associated receptor and ligand genes were screened in CellChatDB by deduplication and the retention of unique values based on 1939 validated molecular interactions (Table S2). We integrated mRNA sequencing data from the TCGA-LIHC and GSE14520 cohorts with prognostic information into a meta-cohort and identified regulatory genes associated with prognosis based on univariate Cox regression. As a result, 20 genes were screened for prognostic associations (Fig. 3A). The two patient cohorts were classified separately using consistent NMF clustering based on the 20 gene expression profiles. The clustering results of three profiles were more stable than the others (Fig. 3B). Three molecular subtypes with different genomic heterogeneities were obtained in the separate cohorts (Fig. 3C). After analyzing the prognostic characteristics of these three molecular subtypes, a significant prognostic difference was observed (Fig. 3D,E): of these three subtypes, cluster C was related to the worst prognosis and cluster A to the best prognosis. A heatmap of the clinical information and gene expression in the meta-analysis cohort was then constructed. The results showed that 113 macrophage-associated receptor-ligand genes were up-regulated in cluster C (Fig. 3F).

### Mutation characteristics and enrichment analysis of molecular subtypes

**Figure 4:**
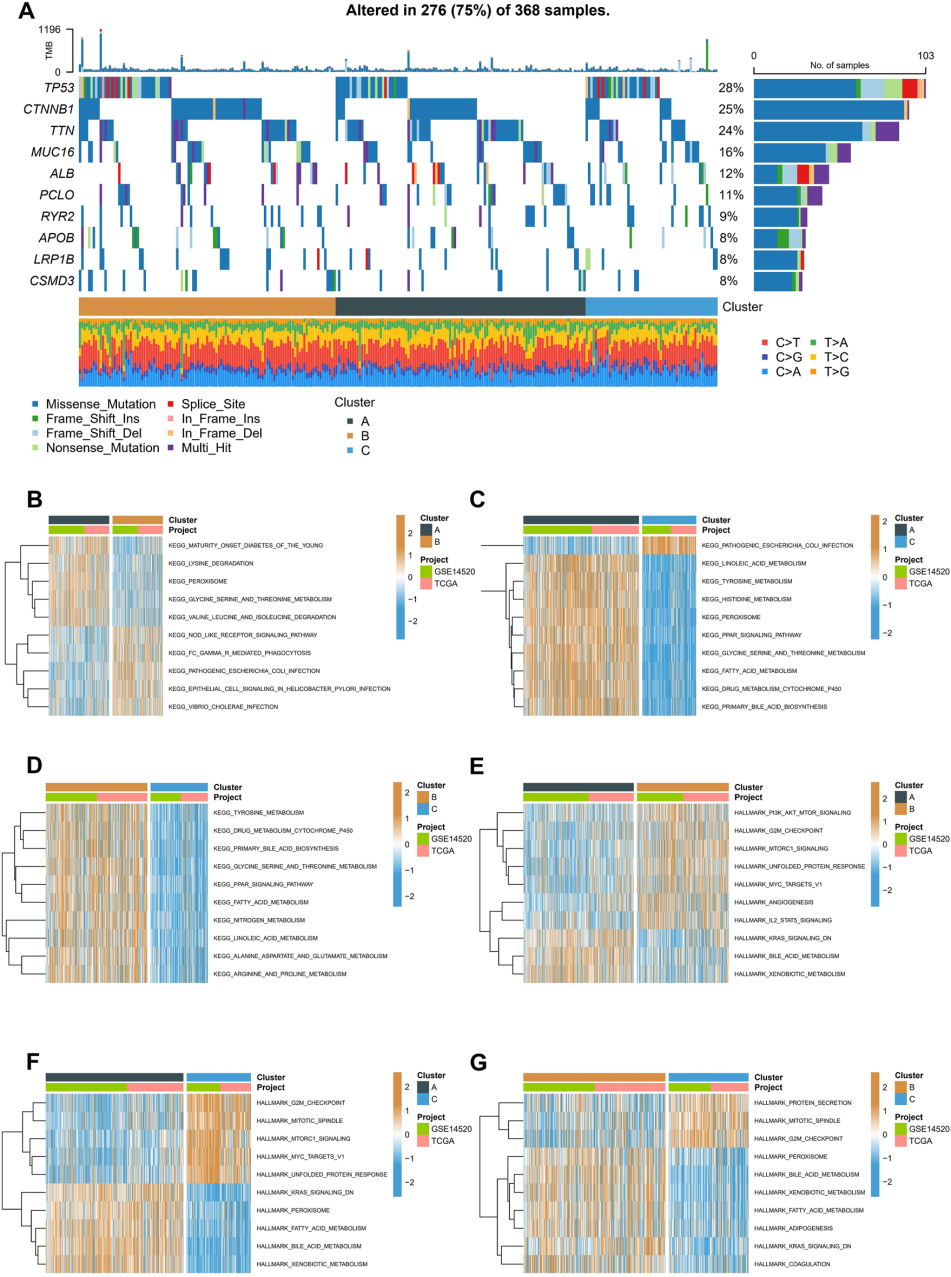
Mutation characterization and enrichment analysis of molecular subtypes. (**A**) Somatic mutation analysis of different molecular subtypes in the TCGA-LIHC cohort. (**B**–**D**) GSVA enrichment analysis of different molecular subtypes based on KEGG gene sets. Cluster A versus Cluster B (**B**), Cluster A versus Cluster C (**C**), Cluster B versus Cluster C (**D**). (**E–G**) Hallmark-based enrichment analysis of different molecular subtypes of GSVA. Cluster A versus Cluster B (**E**), Cluster A versus Cluster C (**F**), Cluster B versus Cluster C (**G**).

SNV mutation data of the TCGA-LIHC cohort were then obtained using Mutect2, the top 10 most frequently mutated genes in each subtype were identified. The mutation frequency of Catenin Beta 1 (CTNNB1), titin (TTN), and other genes in cluster C were found to be significantly lower than those in clusters A and B (Fig. 4A). A GSVA enrichment analysis was then conducted based on different gene sets. In the KEGG gene set, cluster B showed significant enrichment of the NOD_LIKE_RECEPTOR_SIGNALING_PATHWAY and EPITHELIAL_CELL_SIGNALING_IN_HELICOBACTER_PYLORI_INFECTION pathways compared to cluster A (Fig. 4B). Compared to cluster A, cluster C showed substantial enrichment in the PATHOGENIC_ESCHERICHIA_COLI_INFECTION pathway (Fig. 4C). Compared with cluster C, cluster B was significantly enriched in most pathways, such as KEGG_FATTY_ACID_METABOLISM and KEGG_PPAR_SIGNALING_PATHWAY (Fig. 4D). The enrichment of classical oncogenic pathways was also compared. The results showed significant oncogenic pathway differences among the different pathways for each subtype. Interestingly, compared with cluster A, which has the best prognosis, there was a significant inhibition of metabolism-related pathways, such as FATTY_ACID_METABOLISM and BILE_ACID_METABOLISM in cluster C, which has the worst prognosis. These results may assist in explaining the poor prognosis of cluster C (Fig. 4E–G).

### Immunological characteristics of molecular subtypes and differences in immunotherapy/chemotherapy

**Figure 5:**
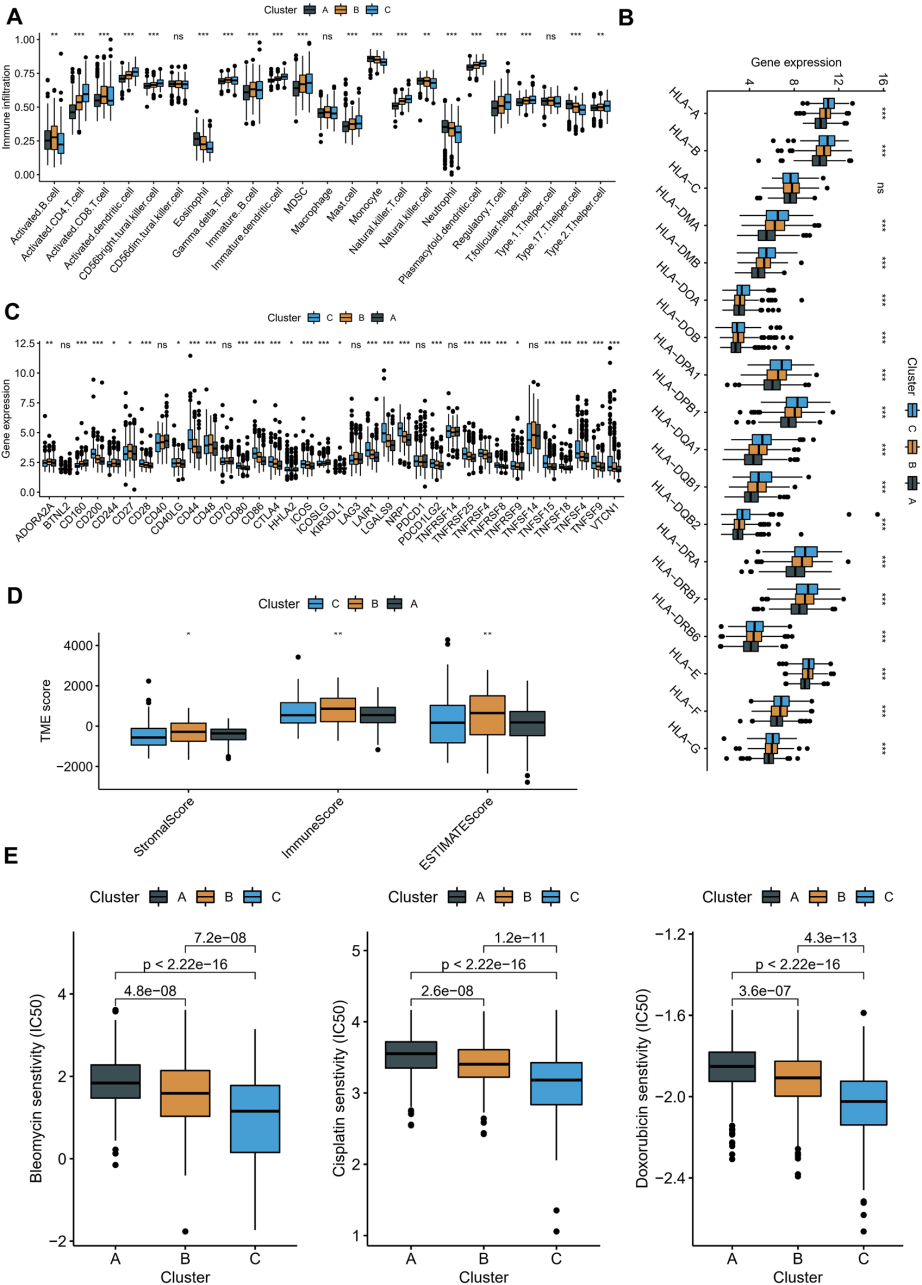
Immunological characteristics of molecular subtypes and analysis of different drug sensitivity. (**A**) Differences in twenty-two immune cell scores among molecular subtypes in the TCGA-LIHC cohort. (**B**,**C**) Expression levels and differential analysis of HLA (**B**) and immune checkpoints (**C**) in three molecular subtypes. (**D**) ESTIMATE algorithm to assess the overall TME landscape across subtypes, including stromal, immune, and estimate scores. (**E**) Drug effectiveness of molecular subtypes. Bleomycin (left), cisplatin (middle), and doxorubicin (right). (ns, not significant; *p* > 0.05; *, *p* < 0.05; **, *p* < 0.01; ***, *p* < 0.001; ****, *p* < 0.0001).

As the molecular subtypes were established based on macrophage-associated receptor ligand genes, it was acknowledged that differences may exist between the immune function and status of the three subgroups. SsGSEA was employed to ascertain the TME status of the various molecular subtypes. The findings revealed that although cluster C had a more significant percentage of activated CD4 + and CD8 + T cells, cluster C had a much higher percentage of regulatory T cells (Tregs) than the other subtypes. Treg cells are a subset of T cells with a solid immunosuppressive impact and are distinguished by a cellular phenotype expressing Foxp3, CD25, and CD4^31^. Tregs can suppress the immune responses of other cells, which suggests that cluster C is in an immunosuppressive state despite the higher infiltration of tumor-infiltrating lymphocytes (TILs), and this may also assist in explaining the poorer prognosis of this cluster (Fig. 5A). Additionally, the expression of common immunological checkpoints and HLA molecules was examined amongst the various molecular subtypes. The results suggested that most HLA and immune checkpoint mRNA levels were higher in cluster C than in the other two clusters (Fig. 5B,C).

Additionally, the TME landscape of the various subtypes was evaluated using the ESTIMATE method, and cluster B was shown to have the highest immune and stromal scores (Fig. 5D). Patients with hepatocellular carcinoma are rarely treated with systemic chemotherapy. They are often treated with local chemotherapy with TACE. As different immune microenvironmental states may suggest different chemotherapy sensitivities, we selected the commonly used drugs recommended by the guidelines for TACE. We calculated the response of each subtype to these drugs. Of these, cluster C was found to have the lowest IC50 (Fig. 5E). These results suggest that cluster C may be more sensitive to TACE.

### Screening of phenotype-related gene sets and functional analysis

**Figure 6:**
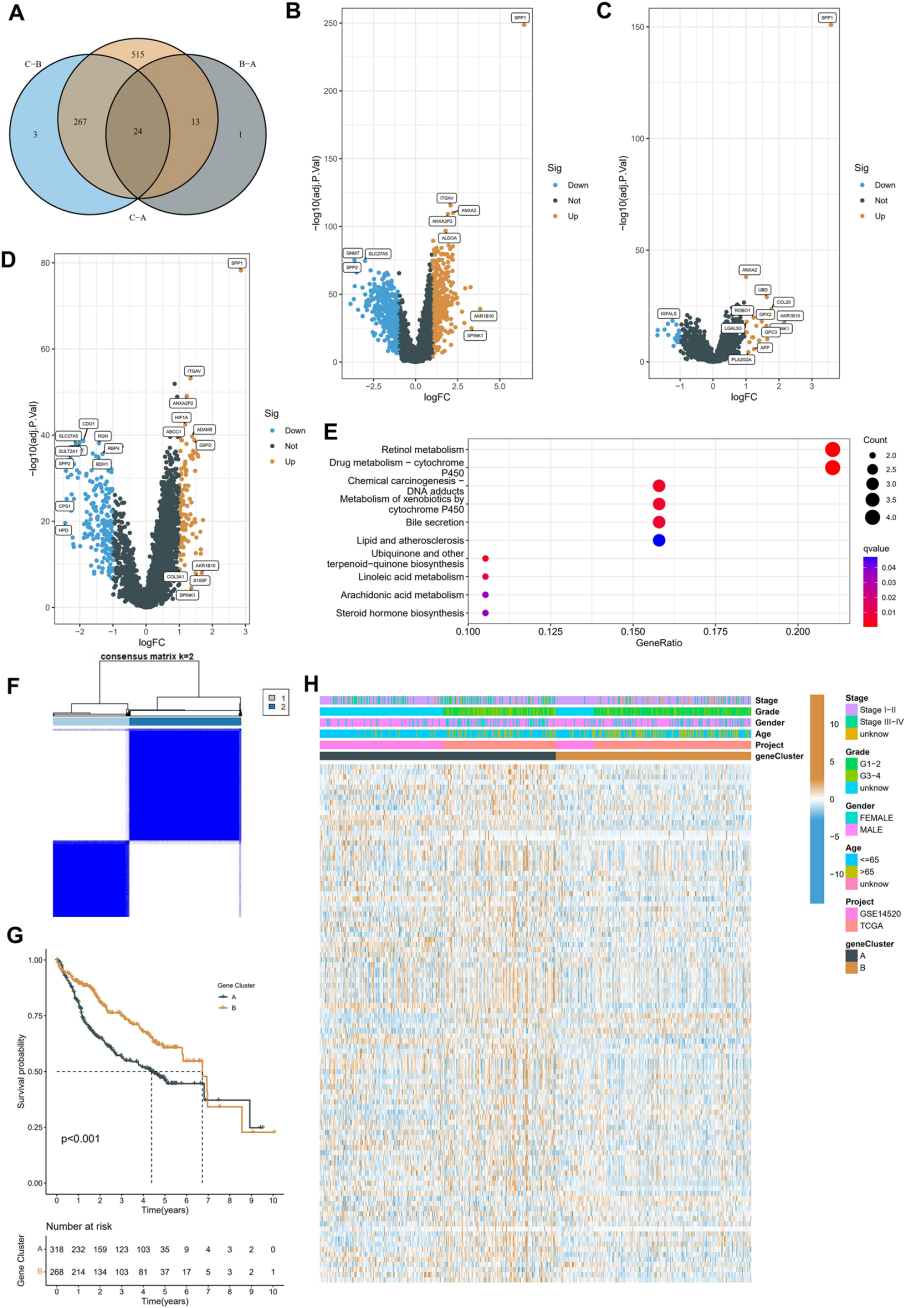
Screening of gene sets and functional analysis. (**A**) Venn diagram of differentially expressed genes between three subtypes. (**B**–**D**) Volcano plots of differentially expressed genes between subgroups. Cluster A versus Cluster B (B), Cluster A versus Cluster C (**C**), Cluster B versus Cluster C (**D**). (**E**) KEGG enrichment analysis of 24 DEGs screened by Venn diagram. **(F)** Molecular subtypes continued to be constructed based on DEGs using the NMF algorithm with the best classification at k = 2. (**G**) Kaplan–Meier survival curves of OS in two clusters. (**H**) Heat map showing the distribution of clinical characteristics of different subtypes.

To understand the causes of the unique immune status and chemosensitivity characteristics of cluster C, we screened for differential genes among the three subtypes using a Venn map, and a total of 24 DEGs were identified (Fig. 6A). Interestingly, the volcanic plot of the different subtypes showed that SPP1 was the most significantly up-regulated gene (Fig. 6B–D). SPP1 is a ligand gene derived from epithelial cells that acts on macrophages. The OPN protein is involved in the synthesis of SPP1 and has a chemotactic effect on macrophages^32^. Similar to the results shown in Fig. 4B–D, our KEGG analysis of these 24 DEGs demonstrated that the enrichment pathway may be related to the biological processes of metabolism (Fig. 6E). We classified the patients into the meta-cohort based on the above DEGs, and the classification was optimal when the K was 2. Two regulatory subtypes (Fig. 6F) were finally identified: cluster A had the best prognosis, and cluster B had the worst prognosis (Fig. 6G). The distribution of the clinical characteristics of the various subtypes was also displayed on a heatmap (Fig. 6H). These results may provide evidence for the prognosis and TME differences associated with different subtypes.

### Construction of risk model

**Figure 7:**
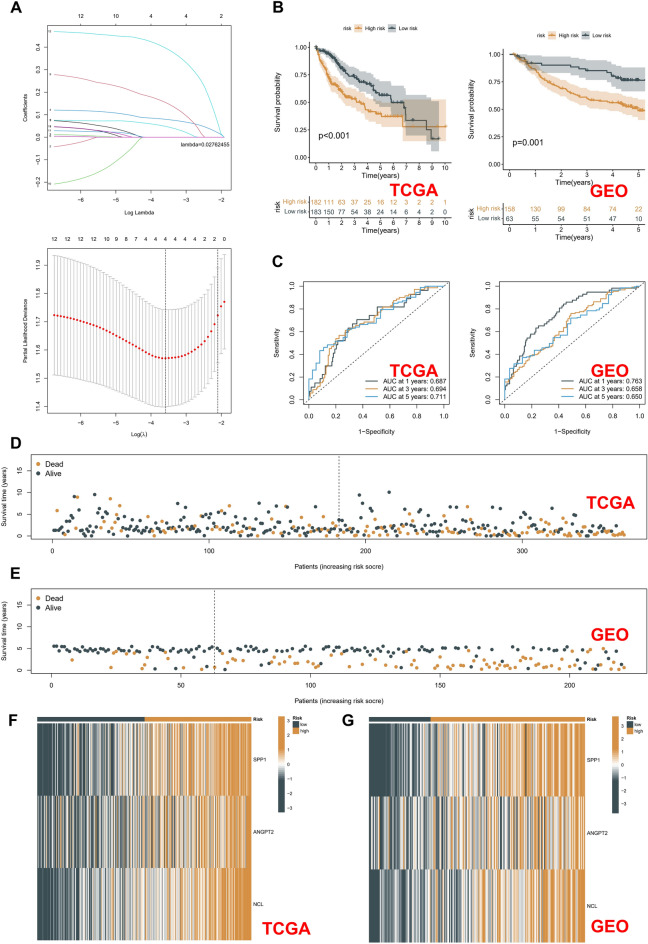
Risk model construction and evaluation. (**A**) The LASSO coefficient profiles (up) and 10-fold cross-validation for variable selection in the LASSO model (down). (**B)** Kaplan–Meier survival curves of OS in different risk groups. (**C**) ROC curves of the risk score in the TCGA-LIHC and GSE14520 cohort. (**D**,**E**) Scatter plot of the risk score in the TCGA-LIHC (**D**) and GEO (**E**) cohorts. (**F**,**G**) Heatmap of three core genes in different risk groups of TCGA-LIHC cohort (**F**) and GSE14520 cohort (**G**).

**Figure 8:**
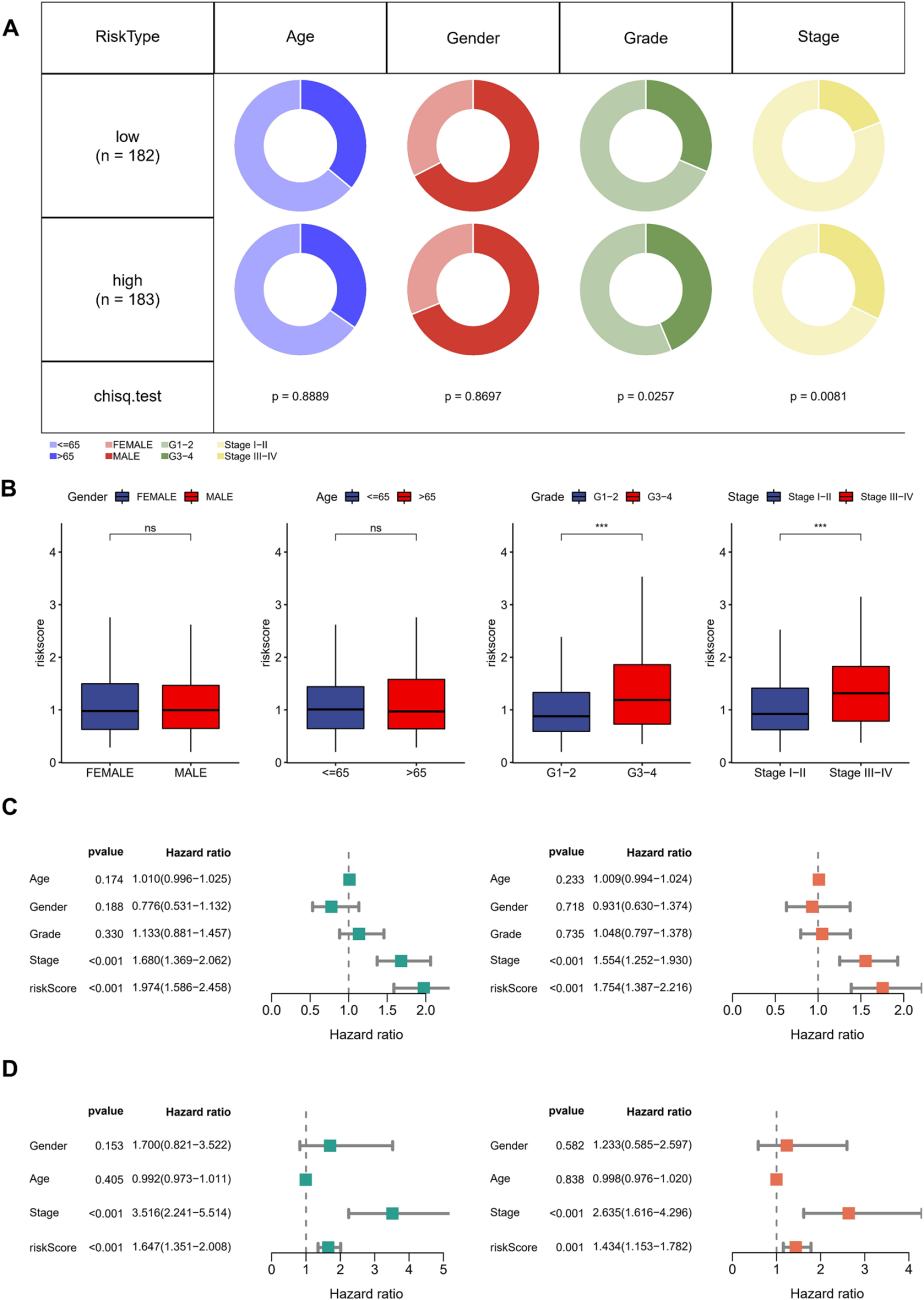
Differences in clinical characteristics of patients with different risk classifications. (**A**) The proportion of patients with different clinical characteristics in the high and low-risk groups. (**B**) Correlation analysis of different clinical characteristics with risk score (Wilcox. Test; *, *p* < 0.05; **, *p* < 0.01; ***, *p* < 0.001; ****, *p* < 0.0001). (**C**,**D)** Univariate and multivariate cox regression analysis of risk score after combining clinical factors. TCGA-LIHC cohort (**C**), GSE14520 cohort (**D**).

The training cohort employed was TCGA-LIHC; the outcome was overall survival time (OS). Subsequently, 113 macrophage-associated receptor genes were initially screened using univariate Cox regression with *p* < 0.05, and redundant genes were then removed using the least absolute shrinkage and selection operator (LASSO) model (Fig. 7A). Furthermore, a multivariate Cox regression analysis was performed to obtain a signature containing three genes. The formula for calculating the risk score for each patient is as follows: Risk score = (0.1034 × expression level of SPP1) + (0.2614 × expression level of ANGPT2) + (0.4932 × expression level of NCL). Based on the median risk score in the TCGA cohort, we divided the TCGA-LIHC patients into high-risk and low-risk groups. The signature was discovered to have a good prognostic value in the TCGA-LIHC and external validation cohort GSE14520, however, the survival probability of high-risk patients was not optimal (Fig. 7B). The ROC curves showed that the risk model has a good predictive efficacy in both the training and validation cohorts (Fig. 7C). In addition, patients with higher risk scores had worse prognoses, as seen by the risk-survival distribution plots of the various cohorts (Fig. 7D–E). The heatmaps of the different cohorts revealed that high-risk patients had significantly higher expression of SPP1, ANGPT2, and NCL in the signatures (Fig. 7F,G). The TCGA-LIHC cohort's risk groups' variances in clinical features were then analyzed. Significant variations in clinical staging and pathological grading were found (Fig. 8A). In addition, patients with high-grade and high-stage HCC had significantly higher risk scores (all *p* < 0.001) (Fig. 8B). After merging the risk score with the clinical parameters, univariate and multivariate Cox regression studies were carried out better to clarify the independent prognostic usefulness of the risk score. The risk score showed a better survival predictive value in both TCGA-LIHC (Fig. 8C) and GSE14520 cohorts (Fig. 8D).

### Immune landscape analysis

**Figure 9:**
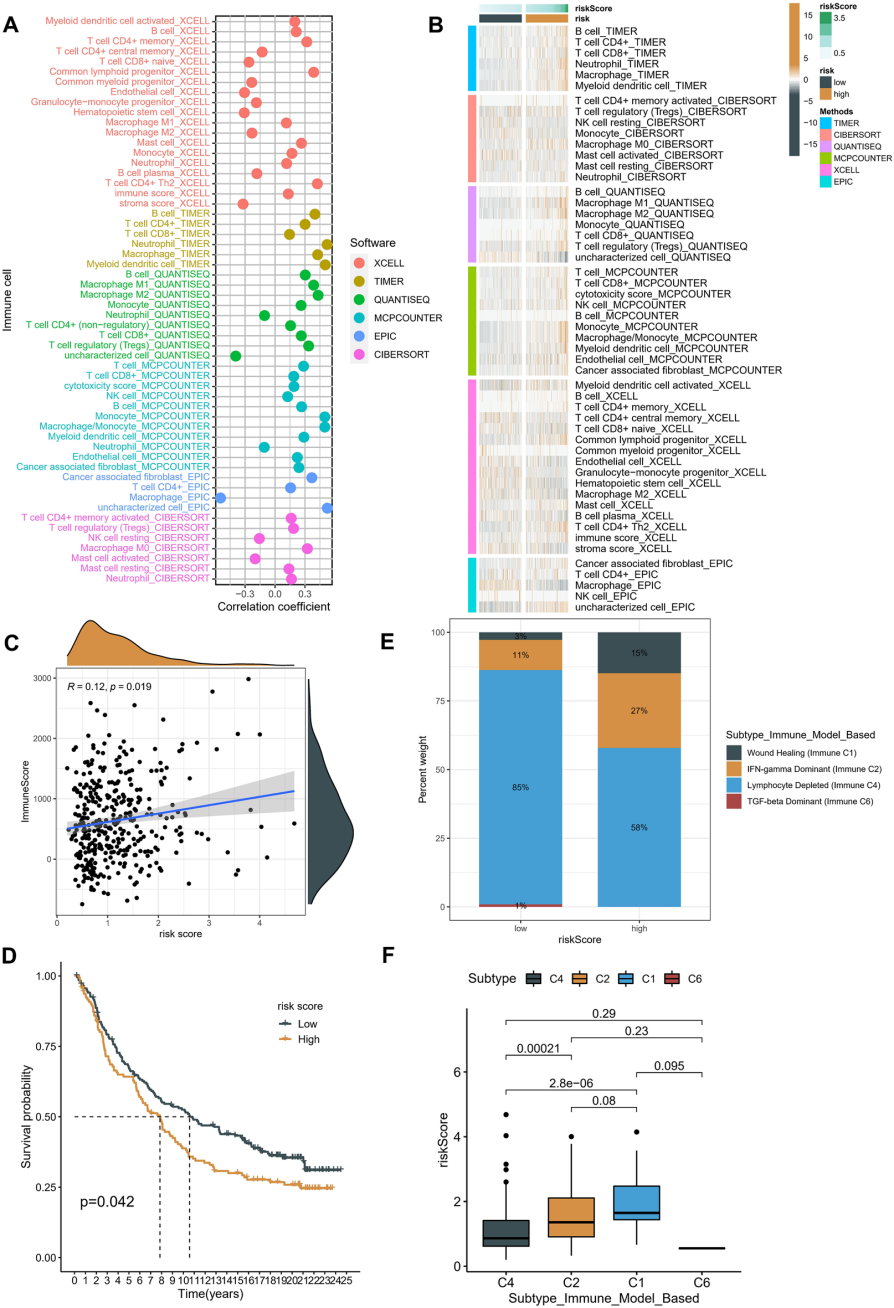
Immune landscape analysis of risk score. (**A**) The correlation coefficient between immune cell infiltration and risk score. (**B**) Heatmap of immune cell infiltration of patients with different risk scores. (**C**) Correlation analysis between immune score and risk score (Spearman Correlation Analysis). (**D**) Patients in the IMvigor210 cohort were divided into high- and low-risk groups after scoring according to the risk score. OS differences were analyzed using Kaplan–Meier survival curves. (**E)** Percentage of patients in different risk groups among the six known immune subtypes. (**F**) Correlation analysis of the risk score with the four immune subtypes: C1, C2, C4, and C6.

We further estimated the infiltration scores of immune cells in several samples using algorithms (such as TIMER, CIBERSORT, QUANTISEQ, MCP counter, XCELL, and EPIC) to examine the relationship between the Risk Score and immunity. A correlation analysis revealed that when the risk score rose, more killer immune cells, such as CD4 + T cells, NK cells, and macrophages, infiltrated the area(Fig. 9A). A heatmap showed that the TME status of the high-risk group was more active than that of other groups (Fig. 9B). ESTIMATE was then used to predict the immune score of patients in the TCGA-LIHC cohort. The relationship with the Risk Score was calculated using Spearman's method. The results revealed that the immune score was significantly and positively correlated with the Risk Score (Fig. 9C).

Two hundred ninety-eight patients from the IMvigor210 cohort receiving anti-PD-L1 therapy were included in this study to further investigate the value of the risk score in predicting the response to immunotherapy. A Kaplan–Meier analysis revealed that the survival rate of high-risk patients were comparatively lower after immunotherapy (Fig. 9D). Thorsson et al. conducted an extensive immunohistochemical analysis of more than 10,000 tumors relating to 33 different cancer types and identified six immune subtypes^26^. After examining the immune subtype distribution in the high-risk group, we discovered that the fraction of lymphocyte-depleted subtypes was dramatically decreased. This finding aligned with the immune cell infiltration score pattern, which rose in the high-risk group (Fig. 9E). The wound-healing subtypes also had a higher risk score (Fig. 9F).

**Figure 10:**
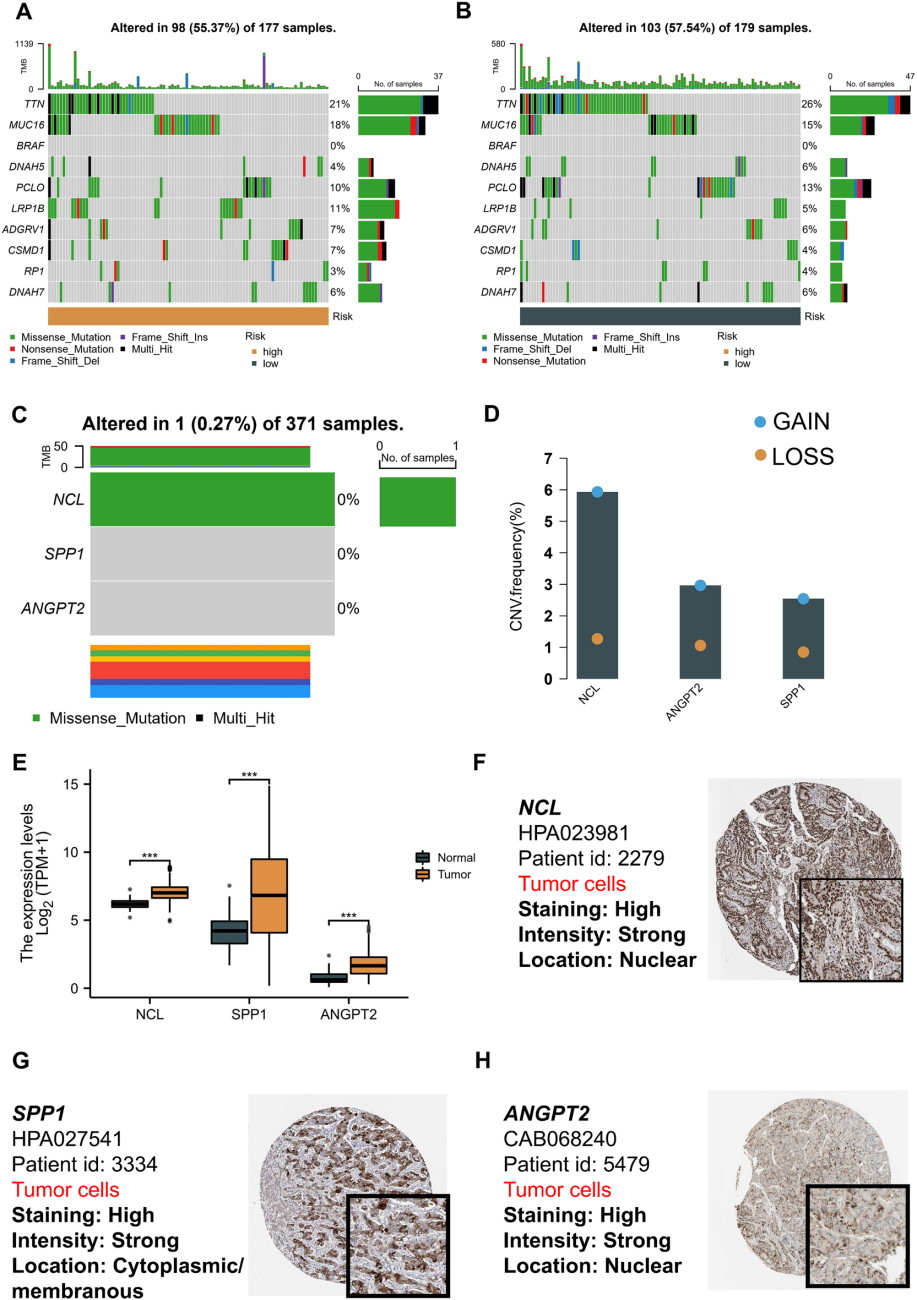
Mutation landscape analysis and tissue expression of core genes. (**A**, **B**) Mutation gene analysis based on whole exome sequencing data in patients with two subtypes of high and low risk. TCGA-LIHC cohort (**A**), GEO cohort (B). (**C**) Mutation analysis of three core genes. (**D**) CNV analysis of three core genes. (**E**) Differential expression analysis of three core genes in the TCGA-LIHC cohort in cancer and para cancer. (**F–H**) Validation of NCL (**F**), SPP1 (**G**), and ANGPT2 (**H**) expression in HCC based on the human protein atlas database.

We performed a mutational gene analysis of the high- and low-risk patients based on whole-exome sequencing data to determine if variations in immunological status between the high- and low-risk groups were brought on by genetic alterations. The Top 10 mutated genes in the two groups did not significantly differ from one another, according to the findings (Fig. 10A,B). Finally, we conducted a mutation analysis of the three essential macrophage receptor ligand genes. The results revealed that only one sample in the TCGA cohort contained a mutation in NCL (Fig. 10C), and no mutations of other genes were observed. All three genes in the signature showed a significant copy number amplification (Fig. 10D). Similarly, compared with paracancerous samples, most of the genes in tumor samples were also significantly up-regulated (all *p* < 0.001) (Fig. 10E). In addition, immunohistochemical results confirmed the high expression of SPP1, ANGPT2, and NCL (Fig. 10F–H).

### Validation of three signature genes

**Figure 11:**
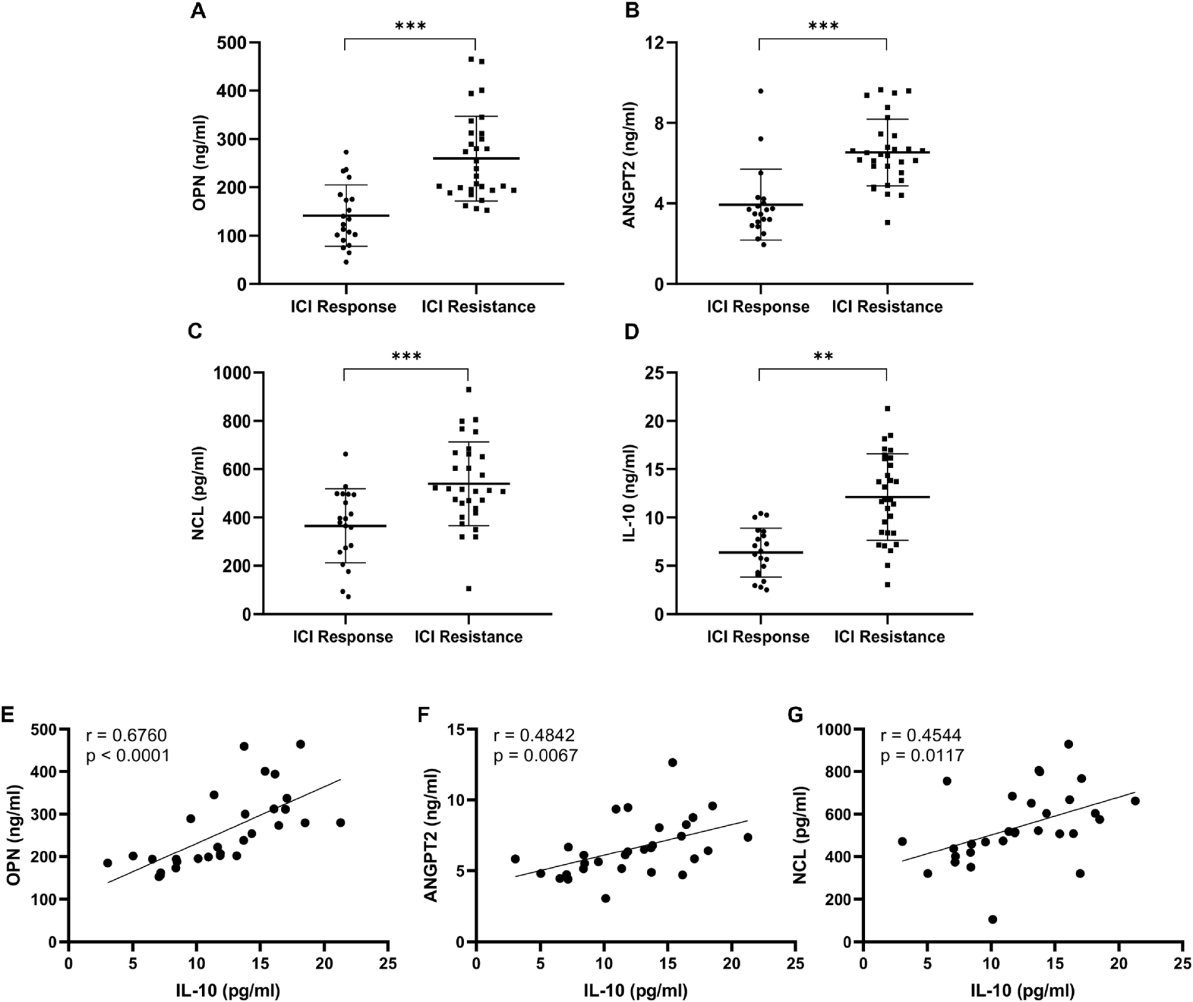
Correlation between signature genes and TME markers in ICI treatment. (**A**–**D**) The levels of OPN (**A**), ANGPT2 (**B**), NCL (**C**), and IL-10 (**D**) in the peripheral blood of patients in the ICI response and ICI resistance groups were measured and then analyzed for differences. (student's t-test. *, *p* < 0.05; **, *p* < 0.01; ***, *p* < 0.001; ****, *p* < 0.0001). (**E**–**G**) Correlation analysis of IL-10 and OPN (**E**), ANGPT2 (**F**), and NCL (**G**) expression levels in peripheral blood of patients with ICI resistance. (Pearson correlation analysis).

In this study, we retrospectively analyzed patients who received immunotherapy based on the iRESIST criteria and divided them into two groups: ICI response and ICI resistance. The baseline data for the two groups of patients are shown in Table S3. ELISA kits were used to detect the levels of OPN and ANGPT2 in serum and NCL in the PBMC lysate. IL-10 is an important factor involved in the induction of M2 macrophage transformation and served as a target for the assay. The results showed that the expression levels of OPN, ANGPT2, NCL, and IL-10 were significantly higher in patients in the ICI-resistant group than those in the ICI-responsive group (Fig. 11A–D). In addition, a correlation analysis showed that OPN (r = 0.6760, *p* < 0.0001), ANGPT2 (r = 0.4842, *p* = 0.0067), and NCL (r = 0.4544, *p* = 0.0117) positively correlated with the expression level of IL-10 (Fig. 11E–G).

**Figure 12:**
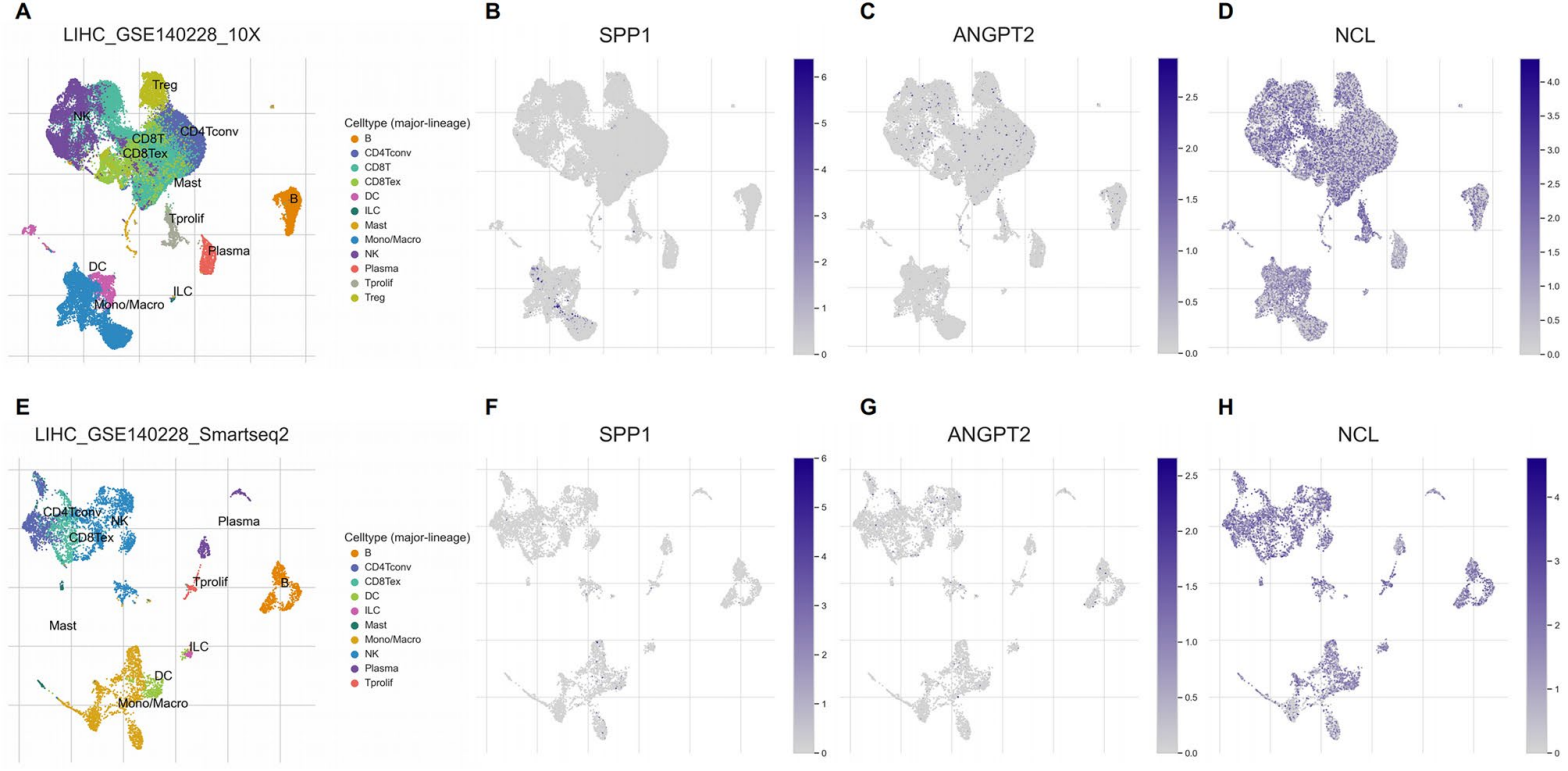
Expression levels of SPP1, ANGPT2, and NCL across different cell types. (**A**–**E**) The scatter plot demonstrates clustering and grouping results of cells within the LIHC_GSE140228_10X (**A**) and LIHC GSE140228 Smartseq2 (**E**). The distribution and expression of target genes SPP1 (**B**–**F**), ANGPT2 (**C**–**G**), and NCL (**D**–**H**) across different cell types.

The GSE140228 dataset contains single-cell RNA sequencing information from patients with HCC aimed at investigating the immune microenvironment of HCC. We analyzed the expression levels of SPP1, ANGPT2, and NCL across different cell types within this dataset, revealing that SPP1 and ANGPT2 are expressed at higher levels in Monocytes/Macrophages cells compared to other cell types (Fig. 12A–H). The mean gene expression levels of these three genes are depicted in Supplementary Figure S1. The results of this gene set validation align with our previous analyses. The above results further confirm that macrophage-associated receptor and ligand signatures are related to the efficacy of immunotherapy, and they are thus valuable for developing and applying individualized treatments.

### Drug-gene interaction and molecular docking analyses of SPP1, ANGPT2, and NCL

**Figure 13:**
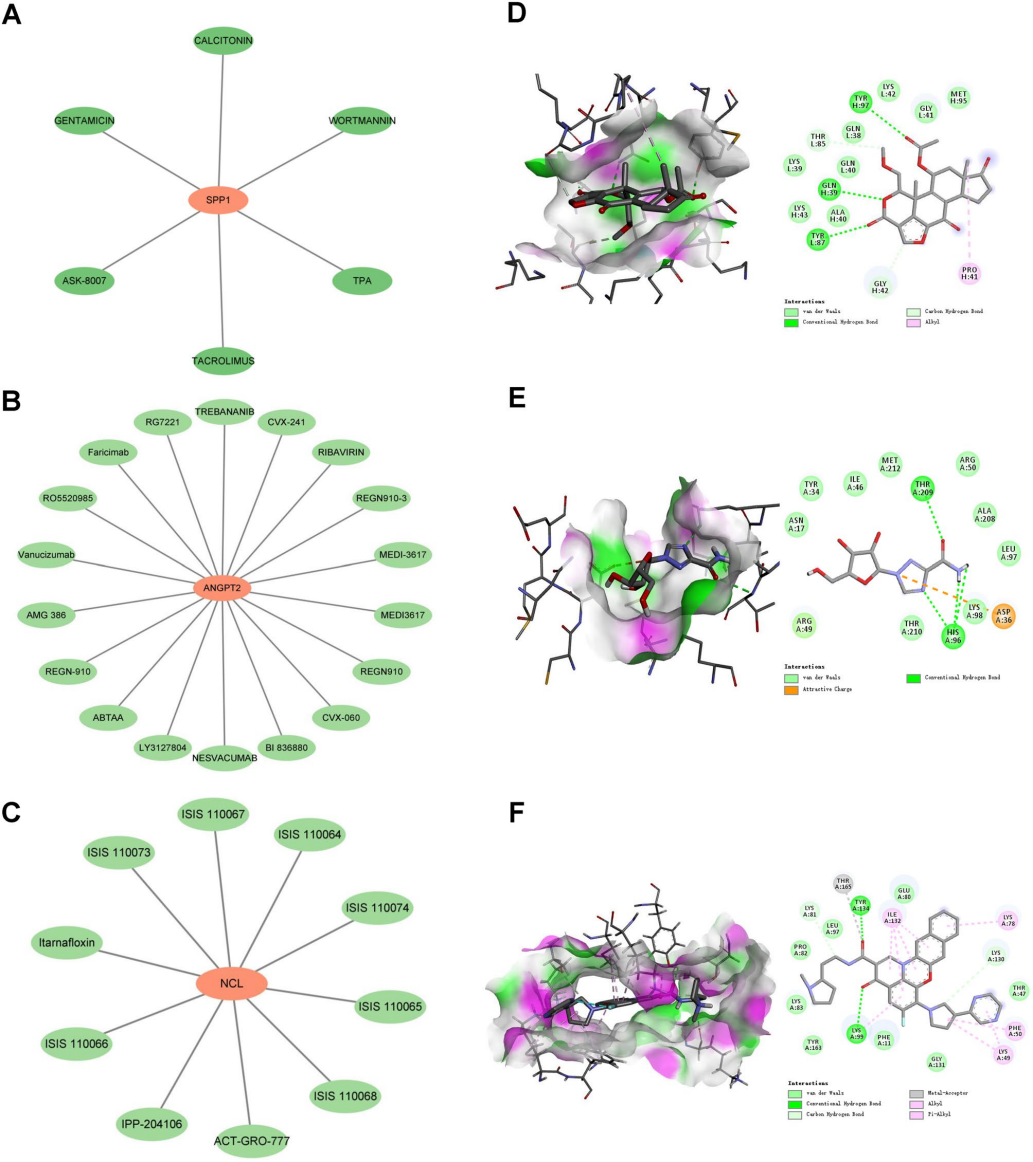
Drug–gene interaction and molecular docking analyses. (**A**–**C**) Drug–gene interaction network of SPP1 (**A**), ANGPT2 (**B**), and NCL (**C**). (**D**–**F**) Molecular docking between three genes and three drugs. SPP1 and Wortmannin (**D**). ANGPT2 and Ribavirin (**E**). NCL and Itarnafloxin (**F**).

The hunt for specific medications for SPP1, ANGPT2, and NCL offers a fresh approach and prospective treatment for treating HCC. The drug-gene interaction network for the three genes is depicted in Fig. 13A–C. It was built using medicines that negatively correlated with the IC50 coefficient. After semi-flexible docking using Autodock-vina, we selected wortmannin, ribavirin, and itarnafloxin to bind to SPP1, ANGPT2, and NCL, respectively, as these had the strongest correlations. The analysis process and the software used (including version information) have been explained in section "Analysis of drug-gene interactions and molecular docking" of Materials and Methods. The percentages and interaction energies of the three compounds are displayed in Table S2, and steady results were obtained when these three pharmaceuticals were coupled with the three genes (Fig. 13D–F), suggesting that they might be employed as novel medications to help cure HCC.

## Discussion

HCC grows and manifests slowly, and people who have the illness in the late stages frequently have few therapy options. Although remarkable advances and breakthroughs in cancer immunotherapies have been made in recent years, the efficacy of immunotherapy varies between individuals. As a result, we examined single-cell transcriptome sequencing data for primary HCC. We relapsed HCC separately in this work to create molecular subtypes based on a gene set connected to macrophage-related receptor ligands. SPP1, ANGPT2, and NCL were identified as novel biomarkers that may influence HCC immunotherapy. Their roles in the immune influence mechanism were investigated to provide a valuable resource for personalized HCC immunotherapy. A single-cell sequencing dataset of primary and relapsed HCC was selected for analysis in this study. After classifying the various cell types, we examined and contrasted the variations in immune cell infiltration between the two sets of samples. DCs and macrophage infiltration were much lower in metastases than in main lesions. Invading tumors, DCs are a diverse collection of innate immune cells that process and deliver tumor-derived antigens to naive T-cells^33^. We hypothesized that HCC metastases would be sensitive to DC-cell-based immunotherapy. We then used CellChat to analyze the number and intensity of intercellular communications in different samples. Macrophages are known to play a central role in intercellular communication in both metastatic and primary lesions, and TAM are a class of macrophages that infiltrate tumor tissues. In contrast to other macrophages, TAMs have specific receptors and ligands; for example, TAM can enhance phagocytosis by binding to extracellular matrix metalloproteinase ligands (e.g., MMP-2, MMP-7, MMP-9, and MMP-12), which in turn promotes tumor growth and metastasis^34,35^.

In addition, TAM can regulate cytokine release and signal transduction pathways by binding to cell surface receptor ligands, thereby affecting tumor cell growth and metabolism. In hypoxic tumor regions, TAM is a crucial source of VEGF-A, a potent mitogen for endothelial cells that binds to VEGFR1/2 in human breast tumors^36^, promoting tumor vascular growth and causing hematologic metastasis. Our analysis also revealed that the secretion of IL-6 was enhanced in metastases. I-6 is known to act by binding to the IL-6 receptor (IL-6R), which triggers receptor-associated Janus kinase (JAK). This stimulates the signal transducer and activator of transcription 3 (STAT3) to initiate downstream signals involved in anti-apoptotic, angiogenic, proliferative, invasive, metastatic, and drug-resistant processes in cancer cells^37–39^. In summary, the innovative analysis of HCC single-cell sequencing data from different sources conducted in this study may provide a new perspective for identifying biomarkers of HCC metastasis.

After conducting a single-cell analysis, we screened 20 genes related to prognosis using a single-factor Cox regression analysis of the extracted macrophage-related receptor ligand gene set. To further explore the differences between the molecular subtypes, we compared multiple perspectives of patient survival, gene mutations, differential gene enrichment, immune status, and common drug sensitivities. The gain-of-function mutation in CTNNB1, which encodes β-catenin, occurs in approximately 35% of human HCC samples^40^. With increased functional activity, Wnt/β-catenin coordinates with several signaling cascades to promote the development of HCC through its downstream effectors^41^. One study analyzed the TCGA-LIHC cohort and found significantly downregulated immune activation and response pathway activity but significant upregulation of pathways related to immune depletion or promotion of tumor growth in CTNNB1-MUT patients^42^. Our results showed that the mutation frequency of CTNNB1 in cluster C was significantly lower than in the other two groups. Based on these results, we believe that patients in cluster C may be more suitable for immunotherapy. We compared the enrichment of the classical oncogenic pathways. Compared to cluster A, which had the best prognosis, metabolic-related pathways (such as FATTY_ACID_METABOLISM and BILE_ACID_METABOLISM) were significantly inhibited in cluster C, which had the worst prognosis. Tumor cells typically increase the fatty acid de novo synthesis rate to produce cell membrane phospholipids and signaling molecules. In addition, some studies have shown that the lipids present in the TME may regulate the infiltration mode of effector CD4 + T cells and determine the results of targeted lipid metabolism therapy for cancer^43^. Therefore, abnormal metabolism in cluster C may be a significant factor in the subgroup's poor prognosis.

We discovered that whereas cluster C had a more significant proportion of activated CD4 + and CD8 + T cells, it also had a much higher proportion of Tregs than the other subtypes. There is growing evidence that Treg cell ablation can activate and improve anticancer immune responses; nevertheless, systemic Treg cell depletion may concurrently result in harmful autoimmunity. Thus, additional research is needed^44^. Lower tumor purity may be linked to higher immune evasion and a worse prognosis. Immune and stromal cells are the two primary non-tumor components of the TME. They can regulate susceptibility to immunotherapy by influencing tumor purity^45^. Most immune checkpoints in cluster C patients were more strongly expressed than those in patients in the other two groups, according to the analysis of the expression of common immune checkpoint genes in this study. We, therefore, speculate that immune checkpoint-based therapy may be more suitable for patients in cluster C.

After considering the intersections of differential genes from the three subgroups, we used a Lasso-Cox regression analysis to identify three key markers associated with macrophage receptor ligands. Macrophages can be classified as M1-type (classically activated macrophages) and M2-type (alternatively activated macrophages), depending on their activation state and roles^46,47^. M2 macrophages primarily release arginase-I, IL-10^48,49^, TGF-β, and other anti-inflammatory cytokines, which reduce inflammation and contribute to tumor growth and immunosuppression^50^. Recent studies have highlighted the pivotal roles of SPP1, ANGPT2, and NCL in regulating the polarization of tumor-associated macrophages (TAMs), offering a new perspective on their potential as targets for cancer therapy. In the context of esophageal squamous cell carcinoma, high expression of SPP1 was found to facilitate the accumulation of M2-like TAMs, correlating with poor prognosis. This process was mediated through the CD44/PI3K/AKT pathway, which promoted M2 polarization alongside the secretion of VEGFA and IL6, thereby driving disease progression. These findings suggest that targeting SPP1 could constitute a therapeutic strategy^51^. Another study, though in a non-tumor setting of myocardial infarction recovery, illuminated the function of nucleolin (NCL) in regulating the immune response by controlling STAT6 and Notch3 pathways to promote M2 macrophage polarization, underscoring NCL's potential in modulating immune reactions^52^. Furthermore, ANGPT2 has been identified as a serum biomarker for immune checkpoint therapy response, with elevated levels associating with reduced survival rates following anti-PD-1 and CTLA-4 therapies and contributing to the development of resistance against anti-angiogenic treatments by enhancing the immunosuppressive properties of macrophages^53^. Collectively, these molecules emerge as central players in orchestrating TAM polarization, implying their potential to refine immunotherapy efficacy and overcome therapeutic resistance. Future in vitro experiments aimed at elucidating the precise mechanisms by which these molecules regulate HCC-related TAM polarization will pave the way for the development of novel therapeutic strategies with a robust evidentiary foundation.

In the CellchatDB database, we screened for receptors/ligands of three key genes. The related receptor-ligand pairs are organized in Table S5. For SPP1, its receptors mainly include CD44 and the integrin family (such as ITGAV_ITGB3, ITGAV_ITGB5, ITGA5_ITGB1, etc.). The interaction between SPP1 and CD44 is thought to inhibit the activation of CD8 + T cells, promoting tumor immune tolerance and immune escape^54,55^. In addition, SPP1 can also synthesize the OPN protein, which combines with various integrin family proteins through the RGD sequence, playing an essential role in the extracellular matrix and cell adhesion processes^56^. OPN has been proven to bind to integrin αVβ3 in liver cancer cells and activate the transcription factor NF-κB, thereby upregulating the transcription of HIF-1α and its downstream gene BMI1 to mediate the maintenance of the stemness phenotype^57^. The interaction between NCL and PTN plays a vital role in tumor progression, especially in tumor angiogenesis, tumor growth, and chemotherapy resistance. An increase in PTN levels enhances the cell surface localization of NCL, which may be related to interactions in the tumor microenvironment^58^. Furthermore, PTN, by binding to NCL on the cell surface, mediates the stimulating effects of various pro-angiogenic factors^59^. The interaction between ANGPT2 (Angiopoietin-2) and TEK (Tie-2 receptor) is important in controlling tumorigenesis. ANGPT2, by binding to the TEK receptor, activates downstream signaling pathways, such as the PI3K/AKT pathway; the ANGPT2/TEK signaling axis is also involved in regulating the tumor microenvironment, including promoting inflammatory responses and attracting immune cells, such as macrophages, which affect tumor behavior by secreting factors that promote tumor growth and metastasis^60^. In addition to TEK, ANGPT2 can also interact with ITGA5_ITGB1, activating the TGFB-ZEB1-GLUT3 signaling pathway, accelerating physiological activities related to glycolytic metabolism in lung cancer cells, thereby causing tumor cells to prefer glycolytic metabolic pathways and accelerating tumor development^61^. IL-10 is an important factor involved in inducing the transformation of M2 macrophages. The three key genes can affect macrophage transformation through various pathways, and the role of IL-10 here is to reflect the state of macrophages. Given that M2 macrophages have unique anti-inflammatory characteristics and IL-10 can help polarize macrophages to the M2 state in conjunction with IL-4^54^, we selected IL-10 as a peripheral blood detection indicator. Whether there is a relationship between the three key genes and IL-10 still requires extensive research.

Our classification of the patients into high- and low-risk groups was based on a risk score created using the three key genes. As the risk score rose, so did the percentage of numerous killer immune cells (including CD4 + T cells, NK cells, and macrophages) infiltrating the area. According to the heat map, the TME of high-risk group was more active than the low-risk group's. This finding suggests that immunotherapy may be more successful in high-risk individuals. The three core genes might be employed in treatment prediction and risk assessment since they may have different effects on the effectiveness of immunotherapy for HCC. The evidence presented above suggests that future research should concentrate on elucidating the mechanisms involved in signal transduction induced by tumor cells and the associated immune cell surface receptors/ligands, which could result in the creation of immune infiltration-targeted personalized therapies for HCC.

New drugs have been introduced to treat HCC in recent years, but immune escape and drug resistance continue. Wortmannin, ribavirin, and itarnafloxin were shown to be medicines targeting these three essential genes and assisting in treating HCC. We discovered networks of targeted therapeutic drugs for SPP1, ANGPT2, and NCL. The outcomes of molecular docking revealed that early drug-gene binding had taken place. The PI3K inhibitor Wortmannin (SL-2052) is potent, permanent, and selective. According to some research, limiting the activation of immune-suppressive pathways and increasing antitumor intrinsic immunity improves immune surveillance of tumors while inhibiting tumor cell migration and proliferation^62^. The recommended treatment for hepatitis C (HCV) is a combination of ribavirin and pegylated interferon (PegIFN), which is beneficial in lowering the development of HCC in patients^63^. A high-level structure called a G-quadruplex is created when DNA or RNA with many repeating G is folded. The G-quadruplex structure is closely linked to DNA replication and is critical for cell division and proliferation^64^. It is anticipated that selective inhibition of cancer cell growth by targeted manipulation of the G-quadruplex structure will emerge, making it a key target for cancer therapy medicines. In this respect, itarnafloxin can interfere with the interaction of nuclear proteins with G-quadruplex structures in ribosomal DNA, and it is a potential tumor growth-inhibiting drug.

This study contains several restrictions. First, our macrophage-related receptor ligand genes were derived from conducting a CellChat analysis; the results were obtained through algorithms, and they thus may deviate from the actual situation. In addition, many indicators reflect macrophage polarization, but we only selected IL-10 in serum for detection; therefore, further in vivo and in vitro experiments are required to verify the correlation between genes in the prediction model and macrophage polarization.

## Conclusions

This research examined single-cell sequencing data from primary and metastatic liver cancer tissues. The innovative extraction of macrophage-related receptor ligand genes showed that they were related to prognosis, and molecular subtypes were constructed based on the information acquired. The different molecular subtypes relate to different immune microenvironment states and drug sensitivities. We discovered SPP1, ANGPT2, and NCL as critical indicators associated with HCC and created a novel HCC prediction model based on the differential genes of the molecular subtypes. We also screened SPP1, ANGPT2, and NCL-based targeted agents to develop a new strategy for enhancing the efficacy of HCC immunotherapy. In addition, we collected the serum of patients with HCC receiving ICI treatment and studied the expression differences of three key genes and their correlations with IL-10 in patients whose responses to ICI treatment varied. We anticipate that these results may help guide the development of targeted prevention and personalized treatment of HCC.

### Supplementary Information

Supplementary Information 1.

Supplementary Information 2.

Supplementary Information 3.

Supplementary Information 4.

Supplementary Information 5.

Supplementary Information 6.

Supplementary Information 7.

## Data Availability

For original data, please get in touch with the corresponding authors.
