# Author Correction: Multi-omics analysis of macrophage-associated receptor and ligand reveals a strong prognostic signature and subtypes in hepatocellular carcinoma

**DOI:** 10.1038/s41598-024-70952-z

**Published:** 2024-08-29

**Authors:** Yulou Zhao, Cong Chen, Kang Chen, Yanjun Sun, Ning He, Xiubing Zhang, Jian Xu, Aiguo Shen, Suming Zhao

**Affiliations:** 1 Department of Interventional and Vascular Surgery, Affiliated Hospital of Nantong University, Nantong, China; 2 Medical School, Nantong University, Nantong, China; 3 The Sixth People’s Hospital of Yancheng City, Yancheng, China; 4 Department of Medical Oncology, Nantong Second People’s Affiliated Hospital of Nantong University, Nantong, China; 5 Cancer Research Center Nantong, Affiliated Tumor Hospital of Nantong University, Nantong, China

Correction to: *Scientific Reports* 10.1038/s41598-024-62668-x, published online 28 May 2024

The original version of this Article omitted an affiliation for Yulou Zhao. The correct affiliations are listed below.

Medical School, Nantong University, Nantong, China.

Department of Interventional and Vascular Surgery, Affiliated Hospital of Nantong University, Nantong, China.

In addition, Yulou Zhao and Cong Chen were omitted as equally contributing authors.

The original article has been corrected.

