# Supplementary figures and images for: Multi-omics analysis of macrophage-associated receptor and ligand reveals a strong prognostic signature and subtypes in hepatocellular carcinoma
Source: Sci Rep. 2024 May 28;14:12163. doi: 10.1038/s41598-024-62668-x (PMC11133315; doi:10.1038/s41598-024-62668-x)

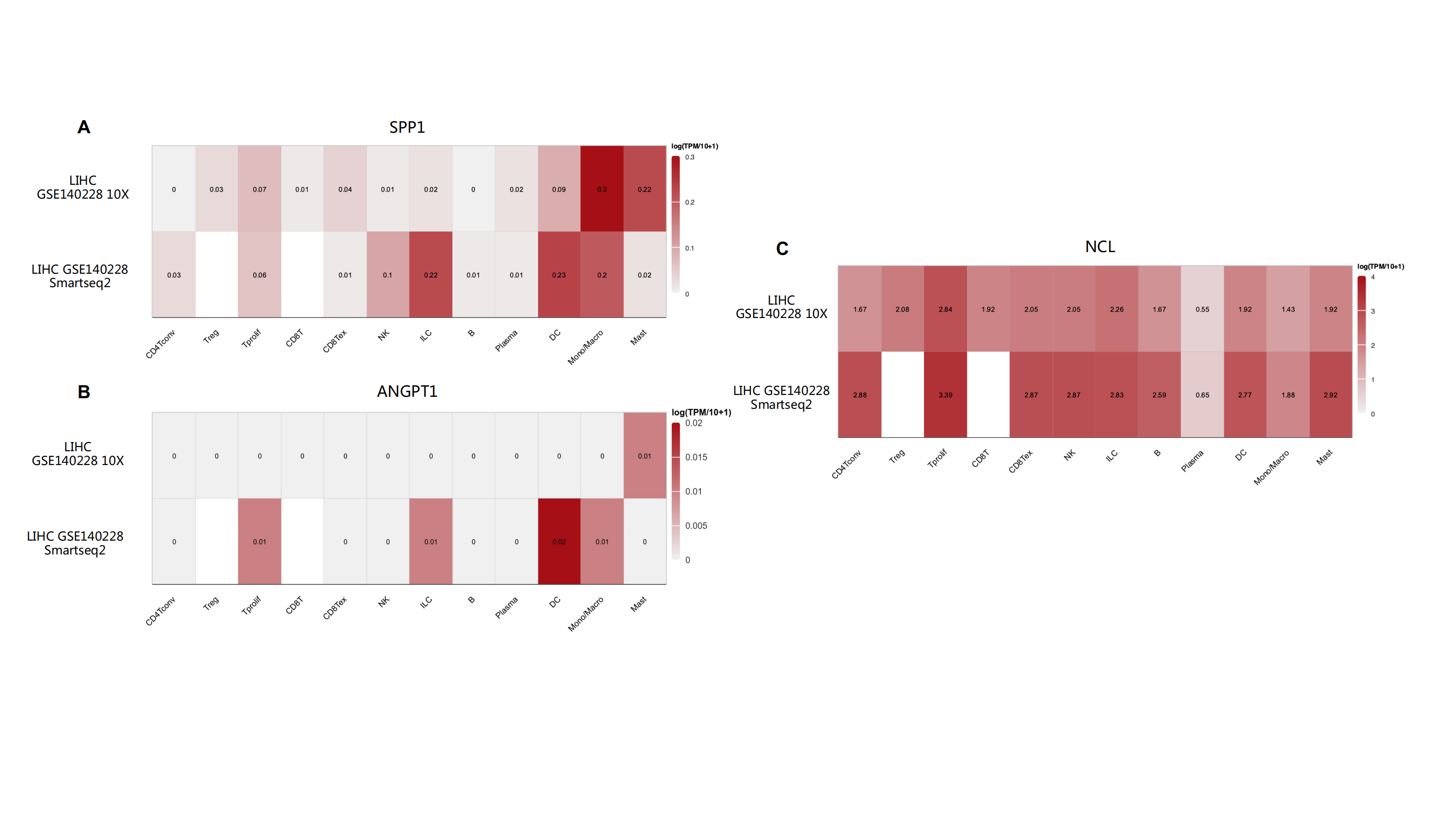

Supplement: Supplementary file 6 — Supplementary Information 6. [file 41598_2024_62668_MOESM6_ESM.tif]
