# Supplementary material for: Multi-omics analysis of macrophage-associated receptor and ligand reveals a strong prognostic signature and subtypes in hepatocellular carcinoma
Source: Sci Rep. 2024 May 28;14:12163. doi: 10.1038/s41598-024-62668-x (PMC11133315; doi:10.1038/s41598-024-62668-x)
Supplement: Supplementary file 7 — Supplementary Information 7. [file 41598_2024_62668_MOESM7_ESM.docx]

**Supplementary Figure and Table Legends**

Fig. S1. Average gene expression level of SPP1 (A), ANGPT2 (B), and NCL (C) in LIHC_GSE140228_10X and LIHC GSE140228 Smartseq2 dataset.

Table S1. Ligand receptor docking parameters.

Table S2. Macrophage-associated receptor and ligand genes.

Table S3. Baseline clinical characteristics of enrolled patients.

Table S4. The fractions and interaction energies of compounds.

Table S5. The relevant receptor-ligand pairs of SPP1, ANGPT2, and NCL.
